# High Human Bocavirus Viral Load Is Associated with Disease Severity in Children under Five Years of Age

**DOI:** 10.1371/journal.pone.0062318

**Published:** 2013-04-30

**Authors:** Baihui Zhao, Xuelian Yu, Chuanxian Wang, Zheng Teng, Chun Wang, Jiaren Shen, Ye Gao, Zhaokui Zhu, Jiayu Wang, Zhengan Yuan, Fan Wu, Xi Zhang, Reena Ghildyal

**Affiliations:** 1 Microbiology Laboratory, Shanghai Municipal Center for Disease Control and Prevention, Shanghai, People's Republic of China; 2 Technical Center for Animal and Plant and Food Inspection, Shanghai Entry-Exit Inspection AND Quarantine Bureau, Shanghai, People's Republic of China; 3 Clinical Laboratory, Shanghai Children's Hospital, Shanghai, People's Republic of China; 4 Centre for Research in Therapeutic Solutions, Faculty of Applied Science, University of Canberra, Canberra, Australia; University Hospital San Giovanni Battista di Torino, Italy

## Abstract

Human bocavirus (HBoV) is a parvovirus and detected worldwide in lower respiratory tract infections (LRTIs), but its pathogenic role in respiratory illness is still debatable due to high incidence of co-infection with other respiratory viruses. To determine the prevalence of HBoV infection in patients with LRTI in Shanghai and its correlation with disease severity, we performed a 3-year prospective study of HBoV in healthy controls, outpatients and inpatients under five years of age with X-ray diagnosed LRTIs. Nasopharyngeal aspirates were tested by PCR for common respiratory viruses and by real time PCR for HBoV subtypes 1–4. Nasopharyngeal swabs from healthy controls and serum samples and stools from inpatients were also tested for HBoV1-4 by real time PCR. Viral loads were determined by quantitative real time PCR in all HBoV positive samples. HBoV1 was detected in 7.0% of inpatients, with annual rates of 5.1%, 8.0% and 4.8% in 2010, 2011 and 2012, respectively. Respiratory syncytial virus (RSV) subtype A was the most frequent co-infection detected; HBoV1 and RSVA appeared to co-circulate with similar seasonal variations. High HBoV viral loads (>10^6^ copies/ml) were significantly more frequent in inpatients and outpatients than in healthy controls. There was a direct correlation of high viral load with increasing disease severity in patients co-infected with HBoV1 and at least one other respiratory virus. In summary, our data suggest that HBoV1 can cause LRTIs, but symptomatic HBoV infection is only observed in the context of high viral load.

## Introduction

Human bocavirus (HBoV) was first discovered in the respiratory secretions of Swedish children with symptoms of acute respiratory infection (ARI) in 2005 [1]. Since its discovery, HBoV has been associated with upper and lower respiratory tract infections (LRTIs) and gastroenteritis worldwide. Recent studies have revealed that three new species, named HBoV2, 3 and 4 are found mainly in the gastrointestinal tract and rarely in the respiratory tract, in contrast to HBoV1 which is a respiratory pathogen [2], [3], [4], [5]. HBoV is classified in the family *Parvoviridae*, which includes small, non-envoloped, icosahedral viruses with 5.3 kb single-strand DNA genome containing three open reading frames (ORFs). The first two sequential ORFs encode non-structural proteins NS1 and NP1, whereas the third downstream ORF encodes two viral capsid proteins, VP1 and VP2 [1].

Evidence is mounting that HBoV1 is an important cause of LRTIs with clinical symptoms including fever, cough, wheezing, dyspnea, abdominal pain and diarrhea [6], [7], 8. Several seroepidemiologic studies have demonstrated that the seropositivity rate for HBoV increases with age, reaching nearly 100% by 6 years of age [9], [10]. Recent data show that HBoV1 is often shed for a long time after primary infection into the respiratory tracts of infants and young children. HBoV1 DNA and anti-HBoV1 antibodies in serum differentiate primary HBoV1 infection from long term post infection shedding [11], with detection of HBoV1 DNA in blood more closely associated with symptoms than positive respiratory samples alone [9].

It is difficult to assess the direct impact of HBoV infection on LRTIs due to its frequent detection in asymptomatic children and co-detection with other respiratory viruses in symptomatic children and few studies have focused on HBoV viral load and disease severity [12], [13]. However, recent studies have implied that high viral load, detection of HBoV alone and viraemia may be associated with RTIs [14], with mono-infection of HBoV being associated with higher viral load than co-infection [15]. Two case reports about severe respiratory disease found a statistical association between HBoV1 and otherwise unexplained LRTIs, in particular, acute wheezing [16], [17], [18]. This suggests that HBoV1 infection, although mainly asymptomatic, has the potential to cause severe disease.

In the current study, we have used molecular detection and viral load measurement in a cohort of healthy, outpatient and inpatient children in Shanghai to determine the association of HBoV viral load with disease severity, in the absence and presence of other respiratory infections.

## Results

### Prevalence of HBoV infection in children in Shanghai, 2009 to 2011

HBoV DNA was detected by real time PCR in 39 out of 554 (7.0%) NPAs from inpatients, 3 out of 29 (10.3%) NPAs from outpatients and 5 out of 195 (2.6%) nasopharyngeal swabs from healthy controls. The clinical symptoms of the 3 HBoV-positive outpatients were fever and cough, without any additional respiratory viruses detected. Among 554 inpatients, other respiratory viruses detected were RSVA (16.2%,90/554), human rhinovirus (HRV) (8.8%, 49/554), Adenovirus (AdV) (6.7%, 37/554), parainfluenza virus-3 (PIV3) (6.1%, 34/554), human metapneumovirus (hMPV) (3.6%, 20/554), RSVB (3.4%, 19/554), Coronavirus OC43/HKU1 (2.3%, 13/554), PIV1 (2.2%, 12/554), Influenza virus A (FluA) (1.6%, 9/554), FluB (1.4%, 8/554), Coronavirus 229E/NL63 (0.9%, 5/554) and PIV2 (0.7%, 4/554). HBoV DNA was also detected by real time PCR in 6 out 13 (46.2%) stool samples and 6 out 11 (54.5%) serum samples from inpatients; of these 4 were from ICU, 1 from department of respiratory medicine and 1 from the neonatal department. The only subtype detected in all samples was HBoV1.

The inpatient demographics and clinical information are summarized in Table 1. The median age of HBoV-positive children was 24 months versus 3 months in HBoV-negative group. Fever, wheezing, dyspnea and diarrhea were not significantly different between HBoV-positive and HBoV-negative groups (Table 1). It is noteworthy that cough and abdominal pain were recorded in more inpatients with HBoV than inpatients without HBoV (89.7% vs 70.5%, p≤0.05 and 7.7% vs 2.5%, p = 0.095 respectively; Table 1).

**Table 1:** Clinical and demographic data of inpatients with and without HBoV.

	HBoV+	HBoV−	p Value
Study population	39	515	
Age			
Median (interquartile)	24M (12M,42M)	3M (25D,12M)	
Sex			
Male (%)	20 (51.2)	308 (59.8)	0.296^▴^
Clinical (%)			
X-ray	39 (100)	515 (100)	
Fever	37 (94.9)	497 (96.5)	0.644^★^
Cough	35 (89.7)	363 (70.5)	0.016^▴^
Wheezing	7 (17.9)	52 (10.1)	0.171^▴^
Dyspnea	17 (43.6)	207 (40.2)	0.677^▴^
Abdominal pain	3 (7.7)	13 (2.5)	0.095^★^
Diarrhea	4 (10.2)	26 (5.0)	0.152^★^

HBoV = human bocavirus; M = month, D = day; ^▴^Mentel-Haenszel χ^2^ test; ^★^Fisher exact χ^2^ test.

**Figure 1:**
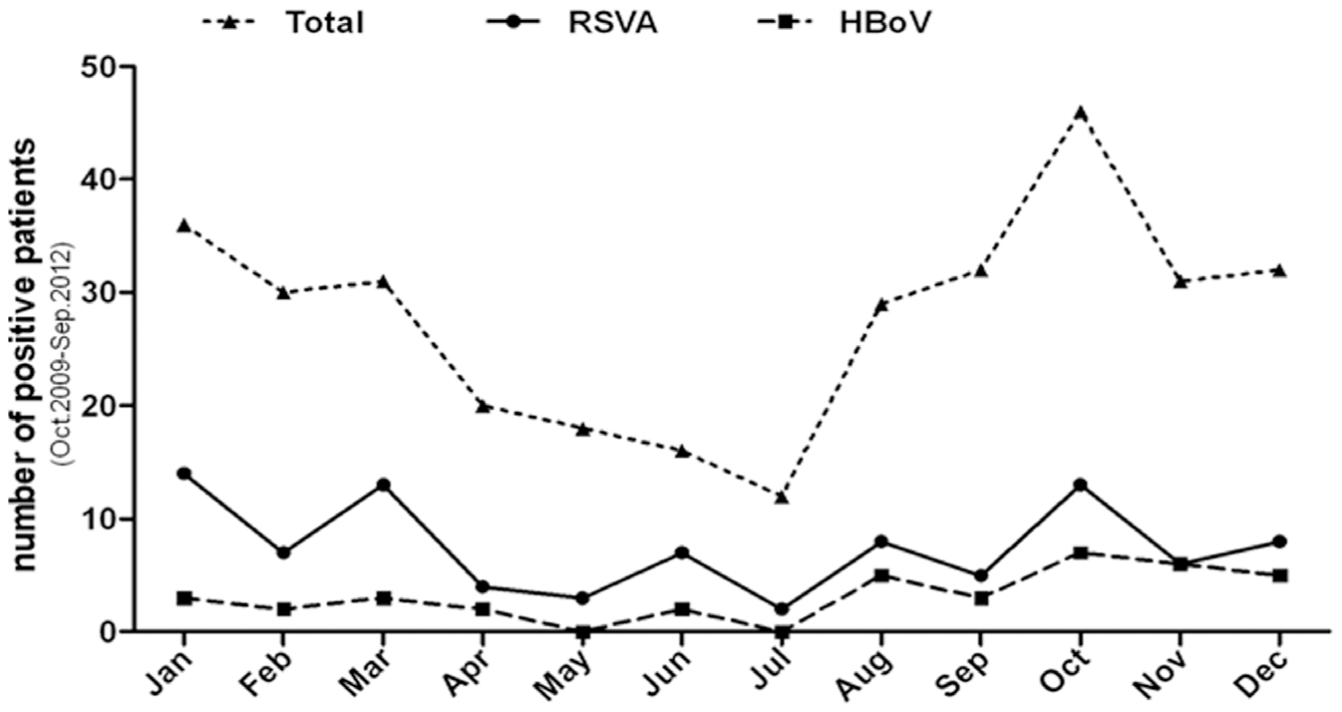
Monthly distribution of HBoV, RSVA and total positive samples during study period (Oct.2009-Sep.2012). NPAs collected from children who were inpatients with LRTI from October 2009 through September 2012 were assayed for 12 common respiratory viruses using the Seeplex RV12 ACE detection Kit. Samples were separately assayed for HBoV by specific real time PCR kit. The incidence of RSVA and HBoV was plotted as the aggregate of positive samples for each month over the study period. The solid line with circles represents RSVA, the dashed line with squares represents HBoV and the dotted line with triangles represents total respiratory virus incidence.

HBoV1 was detected in patient NPAs throughout the course of the study with peak detection in autumn and winter. It was noticeable that HBoV appeared to co-circulate with RSVA (Figure 1), which was the most frequently co-detected virus; however, an extended study with a larger dataset would be required to confirm such a correlation.

### Phylogenetic analysis of HBoV1 circulating in Shanghai, 2009 to 2011

**Figure 2:**
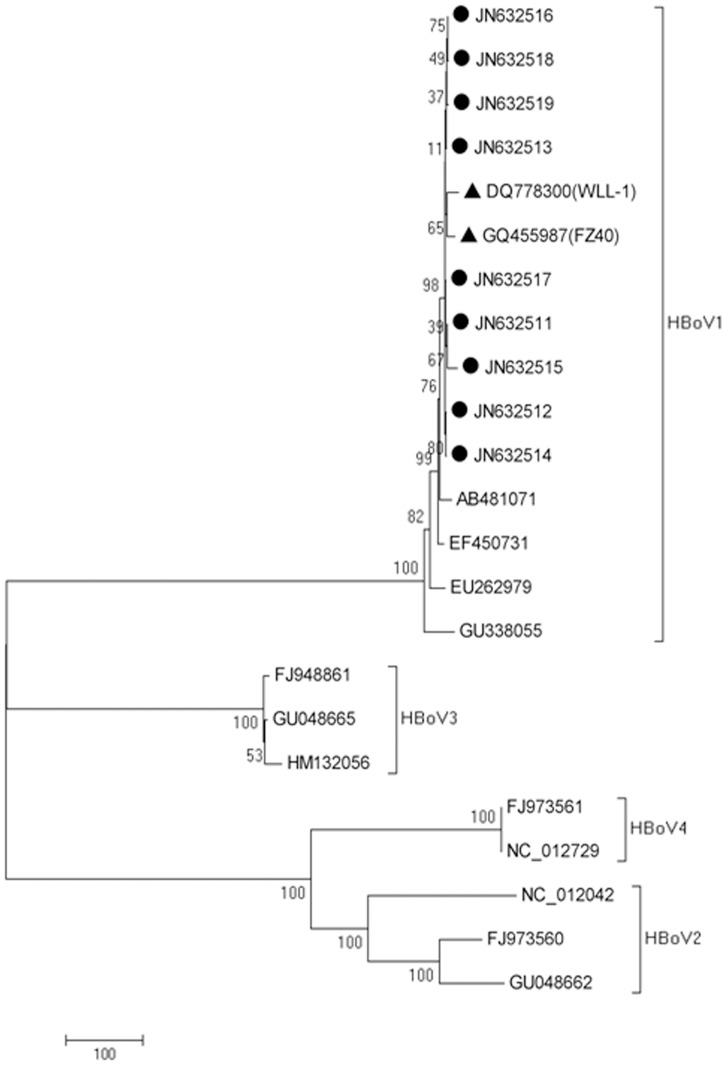
Phylogenetic tree of HBoV sequences. Nine samples with real-time PCR cycle threshold (Ct) value below 24 were chosen for genome sequencing. The nucleotide sequence of HBoV for each sample was PCR amplified, sequenced and analyzed. The phylogenetic tree of HBoV sequences was constructed using Neighbor-joining method by MEGA (5.0 version) software. The bootstrap percentages are given at the node of each branch (1000 replicates). Sequences of 6 different HBoV1 subtype, 3 HBoV2 subtype, 3 HBoV3 subtype, 2 HBoV4 subtype were downloaded from Genbank and used as reference viruses. All the isolates sequenced in this study are indicated by dark circles. The dark triangles represent FZ40 and WLL-1 strains from Fuzhou and Hangzhou, China, respectively.

Nine samples with real-time PCR cycle threshold (Ct) value below 24 were chosen for genome sequencing. Complete genomes were assembled by PCR followed by sequencing of the PCR products; the size of the PCR products was 1099 bp, 1019 bp, 1022 bp, 1101 bp and 1279 bp. Phylogenetic analysis suggested all 9 strains (GenBank accession numbers JN632511-JN632519) had a close genetic relationship to strain FZ40 from Fuzhou and strain WLL-1 from Hangzhou, China (GenBank accession numbers GQ455987 and DQ778300, respectively) (Figure 2). All 9 strains belonged to HBoV1, correlating with the results of subtype real time PCR. Among the 4 subtypes of HBoV, HBoV2 and HBoV4 are grouped together and separate from HBoV1 and HBoV3.

### HBoV co-infection with other respiratory viruses

Of 39 HBoV-positive inpatients, 19 (48.7%) had at least one additional respiratory virus detected (Table 2). The viruses most frequently detected simultaneously with HBoV were RSVA in 9 (47.4%), HRV and hMPV in 3 (15.6%) and FluB in 2 (10.5%) patients. The frequencies of co-detection of one or two additional viruses in patients with HBoV were 89.5% and 10.5%. To assess whether this high frequency of co-detection of other respiratory viruses was a finding specific to HBoV-infected patients, another 41 co-infection samples from LRTI inpatients without HBoV were analyzed. The most frequently detected viruses among these 41 samples were HRV in 25 (61.0%), AdV in 14 (34.1%), RSVA in 13 (31.8%), hMPV and RSVB in 9 (22.0%) patients. The median age of HBoV-positive and HBoV-negative individuals of co-infection patients was 24 months (interquartile range 11–42 months) and 30 months (interquartile range 24–39 months) respectively. There were no significant differences in frequencies of specific respiratory symptoms between the co-infected inpatients with or without HBoV (Table 2). It is noticeable that in the co-infected patient group there were fewer HBoV positive patients than HBoV negative patients with moderate disease (46.3% vs 10.5%, p≤0.05) whereas there were more HBoV positive than negative patients in the group with severe disease (36.8% vs 12.2%, p≤0.05) (Table 2).

**Table 2:** Characteristics of co-infection cohort with and without HBoV.

	HBoV+	HBoV−	p Value
Study population	19	41	
Age			
Median (interquartile)	24M (11M, 42M)	30M (24M, 39M)	
Sex			
Male (%)	9 (47.4)	21 (51.2)	0.7831^▴^
Clinical (%)			
X-ray	19 (100.0)	41 (100.0)	
Fever	16 (84.2)	38 (92.7)	0.2800^★^
Cough	15 (80.0)	31 (75.6)	0.5262^★^
Wheezing	3 (15.8)	4 (9.8)	0.3888^★^
Dyspnea	5 (26.3)	20 (48.8)	0.1035^▴^
Abdominal pain	2 (10.5)	1 (2.4)	0.2332^★^
Diarrhea	2 (10.5)	2 (4.8)	0.3770^★^
Disease Severity (%)			
mild	10 (52.6)	17 (41.5)	0.4225^▴^
moderate	2 (10.5)	19 (46.3)	0.0073^▴^
severe	7 (36.8)	5 (12.2)	0.0332^★^

HBoV = human bocaviruses; M = month; ^▴^Mentel-Haenszel χ^2^ test; ^★^Fisher exact χ^2^ test.

### HBoV viral load is associated with disease severity in children

**Figure 3:**
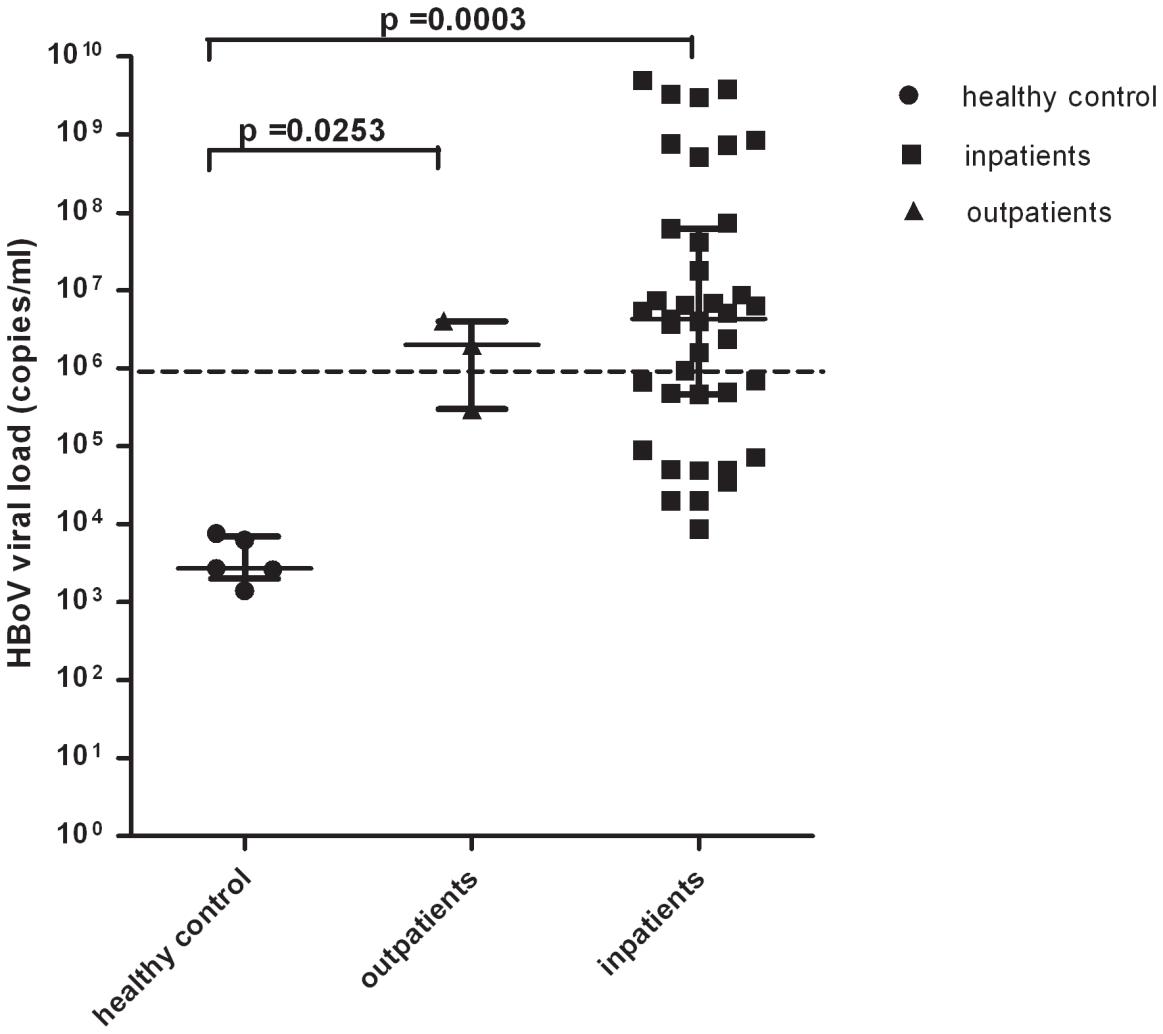
HBoV viral loads in healthy controls, outpatients and inpatients. Healthy controls were randomly chosen from asymptomatic children under the age of five. Outpatient NPAs were collected from children who visited the hospital due to LRTIs but were not hospitalized. Inpatients were children admitted to hospital with LRTIs. The HBoV viral loads (DNA copies per mL) were estimated in 47 HBoV-positive samples (including 5 healthy control, 3 outpatient and 39 inpatient samples) with HBoV real time PCR kit using Roche Light Cycler 480 as described in the text. The positive control (1×1010 copies/mL) provided in the kit was used to generate a standard curve. The viral load (median with interquartile range) in healthy controls (dark circles), outpatients (dark triangles) and inpatients (dark squares) are shown. The differences in HBoV viral load between healthy controls and inpatients, and between healthy controls and outpatients were analyzed by Kruskal-Wallis H χ^2^ test and are shown on the diagram. Significance was accepted at P≤0.05.

Absolute quantification of DNA by real time PCR revealed that the HBoV viral load in HBoV-positive children was highly variable, ranging from 1.4×10^3^ copies/mL to 5.0×10^9^ copies/mL, with a median value of 3.7×10^6^ copies/mL. In order to assess the association between viral load and disease, 39 NPAs of inpatients, 3 NPAs from outpatients without any treatment and 5 nasopharyngeal swabs (NPSs) of healthy controls were chosen. The median viral load of healthy controls, outpatients and inpatients were 2.7×10^3^ copies/mL, 2×10^6^ copies/mL and 5.1×10^6^ copies/mL, respectively (p≤0.05) showing that inpatients and outpatients had higher loads than healthy controls (Figure 3). Interestingly, the median viral load in NPAs from inpatients in whom HBoV was the only virus detected (6.6×10^6^ copies/mL) was not significantly higher than that in inpatients with HBoV co-infection with other respiratory viruses (2.2×10^6^ copies/mL, p = 0.0637). In accordance with previous studies, we chose 1.0×10^6^ copies/mL as the threshold value to differentiate between high and low viral loads [15]. The median viral load of symptomatic children, including outpatients and inpatients, was high whereas the median viral load of asymptomatic children was low.

**Figure 4:**
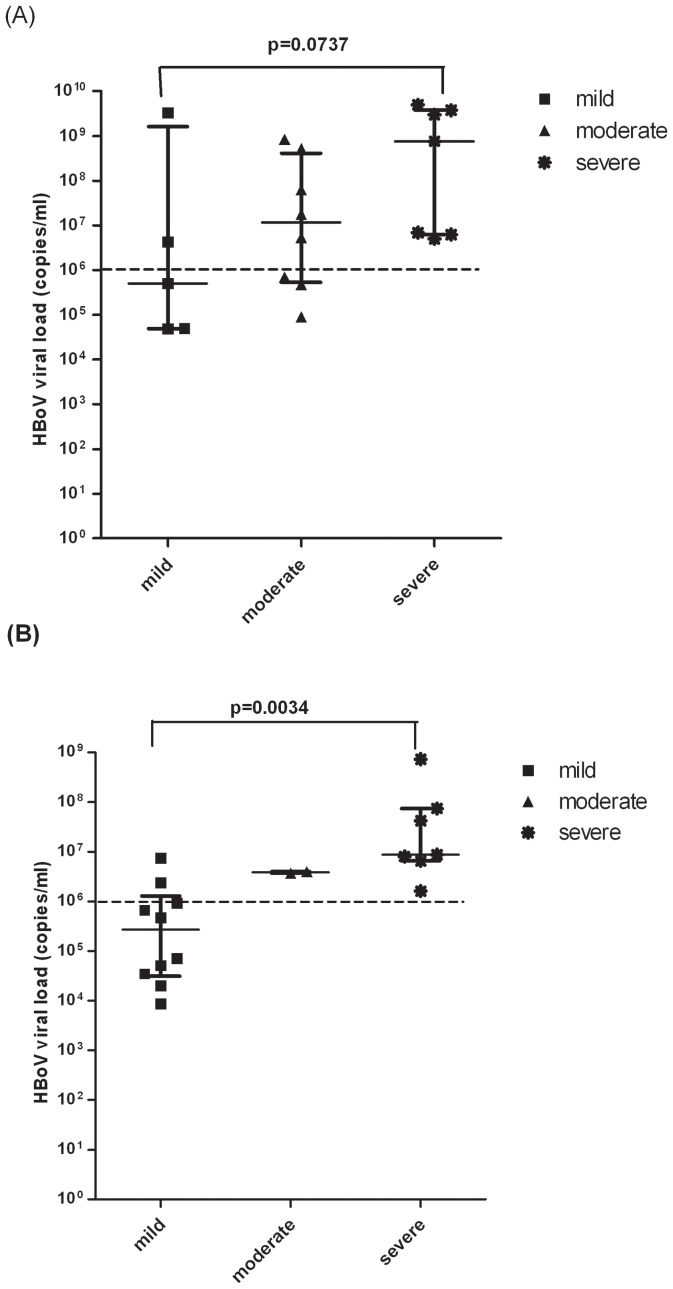
HBoV viral load is associated with disease severity in co-infected patients. Disease severity was assessed according to index of severity (IOS) as described in the text. Patients with IOS value of 0–2 were classified as having mild disease, patients with IOS value of 3–5 were classified as having moderate disease and patients with IOS value of 6–7 were classified as having severe disease. HBoV viral load was measured in 39 HBoV-positive inpatient samples as described in the legend to Figure 3. Patients with viral load of ≥10^6^ copies/mL were termed the high viral load group, and those with viral loads of <10^6^ copies/mL were termed the low viral load group. Statistical differences among the mild, moderate and severe patients were analyzed by Kruskal-Wallis H χ^2^ test and are showed on the diagram. **A.** Median HBoV viral load (with interquartile range) of mild (dark squares), moderate (dark triangles) and severe (dark stars) inpatients with mono-infection with HBoV1. **B.** Median HBoV viral load (with interquartile range) of mild (dark squares), moderate (dark triangles) and severe (dark stars) inpatients with co-infection with HBoV1 and at least one other respiratory virus.

The viral loads of 39 HBoV-positive inpatients with mild, moderate and severe disease in the context of HBoV mono-infection and HBoV co-infection are shown in Figure 4. The median viral loads in mild, moderate and severe patients (5×10^5^ copies/mL, 1.2×10^7^ copies/mL, and 7.6×10^8^ copies/mL, respectively) with only HBoV detected were significantly higher than viral loads in mild, moderate and severe patients (2.7×10^5^ copies/mL, 3.9×10^6^ copies/mL, 8.7×10^6^ copies/mL, respectively) with HBoV co-detected with other respiratory viruses (p≤0.05). In inpatients infected with HBoV1 alone, the median viral loads among mild, moderate and severe group were not significantly different whereas in co-infected inpatients the median vial loads among the three groups were significantly different (Figure 4; also see File S1).

There were two newborn infants, one aged 12 days from the neonatal ward and the other aged 18 days from ICU, whose samples were positive for HBoV only. NPA and stool samples were collected from the 12 day old infant and NPA, stool and whole blood samples were collected from the 18 day old infant. The 12 day old infant had HBoV viral load of 9.0×10^4^ copies/mL in the NPA with no HBoV detection in stool sample, whereas the 18 day old infant had an HBoV viral load of 5.0×10^9^ copies/mL in the NPA with HBoV positive stool and serum samples. The 18 day old child had more severe clinical symptoms than the 12 day old child including fever, dyspnea needing oxygen therapy and diarrhea.

## Discussion

Our study in children under five in Shanghai suggests that HBoV1 may be a causative virus for LRTI symptoms observed in patients; symptomatic children in our study had higher viral load than asymptomatic children, with HBoV1 viral load correlating with disease severity in co-infected children.

To the best of our knowledge, this is the first systematic study of the role of HBoV infection in LRTIs conducted in Shanghai. Previous studies including children with acute respiratory tract infections conducted in Shanghai reported HBoV infection rates of 4.6% to 11.8% [19], [20]. In our study the positive rate of HBoV of NPAs from patients with LRTIs was 7.2% (42/583), consistent with previous studies. Importantly, the present study reported six patients with NPAs, serum and stool being HBoV positive. The median viral load of NPAs for these 6 patients were significantly higher than other HBoV-positive samples (5.1×10^6^ vs 6.3×10^8^, p≤0.05). Detection of HBoV genomic DNA in blood with high viral load in NPAs strengthened the causal link between the virus and disease, which agrees with findings in other studies [9], [14]. The only subtype of HBoV circulating in Shanghai was HBoV1; HBoV2-4 were not detected. Phylogenetic analysis demonstrated that the HBoV1 strain circulating in Shanghai is closely related to the FZ40 and WLL-1 strains reported from Fuzhou and Hangzhou, China. Interestingly, we found that HBoV1 co-circulated, to a large extent, with RSVA in the study period with peaks in autumn and winter. RSVA was the most often detected virus in NPAs followed by HRV and AdV, correlating with viruses co-detected with HBoV1, with 47.4% of co-infected patients being infected with RSVA and 15.6% with HRV.

Although HBoV has been cultured in differentiated human airway epithelial cells [21], isolation of the virus from clinical samples has not been achieved in cell lines, with the result that our knowledge of its replication and pathogenesis is very limited. Recent studies have implied that HBoV DNA can exist episomally in infected human tissues and therefore can likely establish persistent infection in the host [22]. In our study abdominal pain was recorded in more HBoV-positive patients than HBoV-negative patients with 1 out 14 (7.1%) patients with low viral load, and 3 out 25 (12.0%) with high viral load recording diarrhea (p = 0.5453). Although the mechanisms of pathogenesis and routes of infection of HBoV are not yet understood, some information is available for bovine parvovirus (BPV), another member of the genus *Bocavirus* that may have implications for HBoV disease. BPV causes respiratory and enteric disease in calves following initial replication in the tonsils and intestinal epithelium, from where it spreads into the blood, lymphoid tissues and respiratory epithelium [23].

Several seroepidemiologic studies have demonstrated that HBoV infects 40–50% of healthy children aged 6 months to 4 years, after which there appears to be widespread immunity. The present study chose 29 outpatients who presented to hospital for respiratory symptoms and 195 healthy controls retrieved from SCDC Sample Bank to analyze the HBoV positive rate and the viral load. Outpatients and inpatients had high viral load (>1×10^6^ copies/mL) whereas the viral load of healthy controls was low. This result confirms that HBoV can exist in the respiratory tract without causing any symptoms; high viral load is necessary for symptomatic disease.

Further, this study investigated the correlation between HBoV viral load and disease severity in patients with only HBoV infection or HBoV co-infection with other respiratory viruses. It is noticeable that in co-infection, HBoV viral load was associated positively with disease severity (p≤0.05). Given that the most frequently co-detected virus with HBoV was RSVA, which can also cause severe LRTIs in children, the frequency of RSVA in different disease severity groups was analyzed. The percentage of RSVA co-detection with HBoV was not significantly higher than other infectious viruses in all disease severity groups (p value: mild 0.6393, moderate 0.1006, severe 0.0670). In addition, the patients who were negative for HBoV but infected with more than one respiratory virus were mostly grouped with moderate disease compared to those who were positive for HBoV (p≤0.05). Additionally, there were more HBoV-positive patients than HBoV-negative patients (P≤0.05) in the severe disease group. Finally, our data of the two infants with HBoV suggest that LRTIs caused by HBoV can lead to severe and life-threatening disease, confirming reports from other studies 16,18. To the best of our knowledge this is the first report of severe disease in an infant under 1 month of age with only HBoV being detected and our data imply that HBoV1 can infect very young infants.

A few cases of severe disease caused by HBoV have been reported previously, with suggestions that infection may be associated with unexplained LRT symptoms [17], [24]; however the underlying mechanisms are not clear. In this study we found an association between disease severity and HBoV1 viral load in co-infected children; however, our study is limited by the small number of patients and a larger study or meta-analysis is needed to confirm this correlation. It is tempting to speculate that other respiratory viruses which co-infect with HBoV may facilitate HBoV invasion and replication by undermining host immune responses resulting in high levels of HBoV replication facilitating its spread to the blood circulation to cause systemic disease; however this remains to be proven.

## Methods

### Study populations

All patient samples used in this study were collected from patients who presented with respiratory symptoms to Shanghai Children's Hospital from October of 2009 through August of 2012. Patients who were under 5 years of old and diagnosed with bronchiolitis and/or pneumonia by X-ray without underlying conditions were recruited into this study. In all 554 patients were enrolled and their nasopharyngeal aspirates (NPAs) were collected within 3 days of admission to hospital. Among them, 11 patients had NPAs, stool and whole blood samples collected, 2 patients had NPAs and stool samples collected concurrently. 64% (355/554), 10.5% (58/554) and 25.5% (141/554) of patients were inpatients in the respiratory ward, neonatal ward and Intensive Care Unit (ICU), respectively. Clinical information was collected by trained staff and recorded. This study was approved by the Ethical Review Committee of Shanghai Municipal Center for Disease Control and Prevention (SCDC).

In 2012, we randomly collected NPAs from 29 outpatients (under 5 years of age) with LRTIs and without any treatment from Shanghai Children's Hospital. Their clinical information was also recorded. To investigate the prevalence of HBoV in healthy children in Shanghai, 195 nasopharyngeal swabs (NPSs) from healthy children (under 5 years of age) were randomly retrieved from SCDC Sample Bank.

### Samples treatment

All samples were collected and stored in sterile tubes and sent to SCDC microbiology laboratory within 3 hours of collection. All NPAs, stools and whole blood samples were tested for common respiratory viruses, while the NPS samples were tested for HBoV.

1.5 ml sterile saline (0.9%) was added into each tube of NPA and tubes centrifuged at 13000 rpm for 2 minutes; the supernatant was removed and pellet resuspended in 200 µL of Eagle's Minimum Essential Medium. Blood samples were centrifuged at 3000 rpm for 5 minutes; plasma was isolated and kept at −70°C. 3 ml sterile saline (0.9%) was added into each tube with stool samples, the mixture was vigorously vortexed for 5 minutes, and centrifuged at 13000 rpm for 5 minutes. The supernatant was aspirated and stored at −70°C.

### Nucleic acid extraction and first strand cDNA synthesis

Total nucleic acid of each sample (200 µl) was isolated by the MagNA Pure LC 2.0 (Roche, Switzerland) using MagNA Pure LC DNA Isolation Kit (Roche, Germany) following manufacturer's recommendations. 60 µl of eluted total nucleic acid was used as template for PCR. 554 NPAs, 11 serum and 13 stool samples were used for reverse transcription using ReverAid First Strand cDNA Synthesis Kit (Fermentas, CA) on GeneAmp PCR system 9700 (AB, Singapore) according to the manufacturer's recommendations.

### Multiplex PCR for 12 respiratory viruses

All 578 first strand cDNAs were used for multiplex PCR for 12 common respiratory viruses using Seeplex RV12 ACE detection Kit (Seegene, Germany). The viruses included influenza A (FluA), influenza B (FluB), respiratory syncytial viruses A (RSVA), and B (RSVB), human metapneumovirus (hMPV), human parainfluenza viruses 1, 2 and 3 (PIV-1,2,3), adenovirus (AdV), human rhinovirus A/B (HRVA/B), human coronavirus 229E/NL63, human coronavirus OC43/HKU1. PCR conditions were as per the manufacturer's protocols and performed on GeneAmp PCR system 9700 (AB, Singapore). Positive and negative controls provided by the company were included in each test.

### Real-time PCR for HBoV subtypes and viral load

Real time PCR for HBoV subtypes was performed in all samples using previously published primers and probes for HBoV1-4 [25]. PCR was performed in a volume of 20 µl using Roche Light Cycler 480 (Roche, Switzerland) and PCR optical 96-well reaction plate (Roche, Switzerland). Each reaction consisted of 10 µl Premix EX Taq (Takara, Japan), 0.4 µl each of sense and antisense primers (10 µM), 0.8 µl probe (20 µM), 6.4 µl DEPC-treated water and 2 µl of DNA template for a final volume of 20 µl. The PCR mix was denatured at 95°C for 30 seconds (s), followed by amplification consisting of 40 cycles of 15 s at 95°C and 1 minute at 60°C. Positive and no-template controls were included in each run. Plasmids containing the target genes were used as positive controls for all four HBoV subtypes and were constructed by Bio-Sune Bio-tech company.

Real time PCR for HBoV viral load was performed in 39 HBoV-positive samples using HBoV real time PCR kit (Shanghai ZJ Bio-Tech Co., Ltd., People's Republic of China) using Roche Light Cycler 480 (Roche, Switzerland). Positive, negative and no-template controls provided in the kit were included in each test. The positive control (1×10^10^ copies/ml) provided in the kit was used to generate a standard curve. The concentration of each detected sample was then calculated automatically according to standard curve by Roche Light Cycler 480.

### PCR for complete genome sequence

The complete genome of HBoV1 is 5299 nucleotide (nt) long; 5 primer pairs were designed to amplify 5 overlapping fragments, covering the whole genome (see Table 3 for primer sequences), and synthesized by Bio-Sune Bio-tech company (Shanghai, China). The conserved and variable regions of the HBoV1 genome were identified by aligning 13 HBoV1 sequences downloaded from Genbank. Primer selection was aided by Lasergene PrimerSelect 7.0.1 software (DNASTAR, inc., CA) for maximum homology. The primer pairs were specific for HBoV1, with no homology found to any other sequences in GenBank. Premix Ex Taq Version 2.0 (Takara, Japan) was used to perform PCR in a volume of 50 µl using GeneAmp PCR system 9700 (AB, Singapore) and optical 96-well reaction plate (AB, Singapore). Each reaction consisted of 25 µl Premix EX Taq, 1 µl each of sense and antisense primers (20 µM), 5 µl HBoV DNA, and molecular grade water to a final volume of 50 µl. PCR consisted of 35 cycles of 15 minutes at 94°C; 30 s at 94°C, 30 s at 45–55°C, 1 minute at 72°C followed by 10 minutes at 72°C. The PCR products were visualized by electrophoresis on a 2% agarose gel containing 0.5 µg/ml ethidium bromide in Tris-borate buffer (pH 8.0). Positive PCR products were purified and sequenced by Bio-Sune bio-tech. Sequences were spliced *in silico* using Lasergene SeqMan 7.0.1 software (DNASTAR, inc., CA). The phylogenetic tree of HBoV sequences was constructed using neighbor-joining by MEGA (5.0 version) software. 6 sequences of HBoV1, 3 HBoV2 sequences, 3 HBoV3 sequences and 2 HBoV4 sequences were downloaded from Genbank and used as the reference viruses in the construction of the tree.

**Table 3:** Primers sequence for HBoV1 genome sequencing.

Complete genome (FZ1/GQ455988)	Sequence(5′-3′)	Position*
Fragment1	**F1**-GCCGGCAGACATATTGGATTCC	1
	**R1-**CTACCTCAGGAAGATGTTC	1099
Fragment2	**F2**-CCTAGATAACGAAGTCATTC	1041
	**R2-**AATCAGTGCAGTATCCGTTTTC	2059
Fragment3	**F3**-AAACTCATTTCCTCTTGG	2001
	**R3-**CAGCAGCAGAAAGCATTTC	3022
Fragment4	**F4**-GTACCGTAGACACTTAGCTAATG	2962
	**R4-**TCTGTAGCATTGCCTCCAG	4062
Fragment5	**F5-**CTTTTTCAATATGGATATATTCC	4001
	**R5**-TGTACAACAACAACATTAAAA	5279

* Position is defined by strain FZ40 (GeneBank NO. GQ455987) as target gene.

### Disease severity

All patients recruited into this study were admitted for LRTIs and were diagnosed with bronchiolitis and/or pneumonia. Disease severity was assessed according to index of severity (IOS) [26], based on potential of hydrogen (PH), partial pressure CO_2_ (PCO_2_), partial pressure O_2_ (PO_2_), need for hospitalization, oxygen therapy and positive airway pressure, not including length of hospital stay. Patients with an IOS value of 0–2 were classified as having mild disease, patients with an IOS value of 3–5 were classified as having moderate disease and patients with an IOS value of 6–7 were classified as having severe disease.

### Statistical analysis

Statistical analysis was performed using the Epi Info software (version 3.5, CDC, USA), and graphs were prepared using Prism Graphpad v5. Mentel-Haenszel, Kruskal-Wallis χ^2^ or Fisher's exact χ^2^ test were used to assess the significance; p value ≤0.05 was considered statistically significant for all tests.

## Supporting Information

File S1 **Supporting Information for** **Figure 4** **.** Viral load (a) and descriptive statistical analysis (b) is presented for study subjects infected with HBoV1 and one other virus (1) or with HBoV1 alone (2).
(DOC)
