# Supplementary material for: High Human Bocavirus Viral Load Is Associated with Disease Severity in Children under Five Years of Age
Source: PLoS One. 2013 Apr 30;8(4):e62318. doi: 10.1371/journal.pone.0062318 (PMC3640090; doi:10.1371/journal.pone.0062318)
Supplement: File S1 — Supporting Information for Figure 4 . Viral load (a) and descriptive statistical analysis (b) is presented for study subjects infected with HBoV1 and one other virus (1) or with HBoV1 alone (2). (DOC) [file pone.0062318.s001.doc]

1. **HBoV1 patients co-infected with another virus**

a) Raw data

| Viral load(copies/mL) | Type(2-mild;3-moderate;4-severe) |
| --- | --- |
| 35000 | 2 |
| 930000 | 2 |
| 2400000 | 2 |
| 7400000 | 2 |
| 20000 | 2 |
| 72000 | 2 |
| 51000 | 2 |
| 670000 | 2 |
| 8600 | 2 |
| 470000 | 2 |
| 4000000 | 3 |
| 3700000 | 3 |
| 8700000 | 4 |
| 1600000 | 4 |
| 42000000 | 4 |
| 6500000 | 4 |
| 8000000 | 4 |
| 74000000 | 4 |
| 730000000 | 4 |

**b) Descriptive Statistics for Each Value of Crosstab Variable**

| **Type** | Obs | Total | Mean | Variance | Std Dev | Minimum | 25% | Median | 75% | Maximum | Mode |
| --- | --- | --- | --- | --- | --- | --- | --- | --- | --- | --- | --- |
| **2** | 10 | 12056600 | 1205660 | 5.28E+12 | 2298019 | 8600 | 35000 | 271000 | 930000 | 7400000 | 8600 |
| **3** | 2 | 7700000 | 3850000 | 4.5E+10 | 212132 | 3700000 | 3700000 | 3850000 | 4000000 | 4000000 | 3700000 |
| **4** | 7 | 8.71E+08 | 1.24E+08 | 7.2E+16 | 2.68E+08 | 1600000 | 6500000 | 8700000 | 74000000 | 7.3E+08 | 1600000 |

**b) Mann-Whitney/Wilcoxon Two-Sample Test (Kruskal-Wallis test for two groups)**

Kruskal-Wallis H (equivalent to Chi square) =11.3707

Degrees of freedom =2

P value = 0.0034

1. **Patients infected with HBoV1 only**

a) Raw data

| Viral load(copies/mL) | Type(2-mild;3-moderate;4-severe) |
| --- | --- |
| 49000 | 2 |
| 3300000000 | 2 |
| 4300000 | 2 |
| 500000 | 2 |
| 50000 | 2 |
| 480000 | 3 |
| 5400000 | 3 |
| 63000000 | 3 |
| 18000000 | 3 |
| 850000000 | 3 |
| 520000000 | 3 |
| 90000 | 3 |
| 700000 | 3 |
| 6300000 | 4 |
| 3800000000 | 4 |
| 760000000 | 4 |
| 6900000 | 4 |
| 5100000 | 4 |
| 3000000000 | 4 |
| 5000000000 | 4 |

**b) Descriptive Statistics for Each Value of Crosstab Variable**

| **Type** | Obs | Total | Mean | Variance | Std Dev | Minimum | 25% | Median | 75% | Maximum | Mode |
| --- | --- | --- | --- | --- | --- | --- | --- | --- | --- | --- | --- |
| **2** | 5 | 3.3E+09 | 6.61E+08 | 2.18E+18 | 1.48E+09 | 49000 | 50000 | 500000 | 4300000 | 3.3E+09 | 49000 |
| **3** | 8 | 1.46E+09 | 1.82E+08 | 1.05E+17 | 3.23E+08 | 90000 | 590000 | 11700000 | 2.92E+08 | 8.5E+08 | 90000 |
| **4** | 7 | 1.26E+10 | 1.8E+09 | 4.4E+18 | 2.1E+09 | 5100000 | 6300000 | 7.6E+08 | 3.8E+09 | 5E+09 | 5100000 |

**b) Mann-Whitney/Wilcoxon Two-Sample Test (Kruskal-Wallis test for two groups)**

Kruskal-Wallis H (equivalent to Chi square) = 5.2142

Degrees of freedom =2

P value = 0.0737
